# Ecological apparency, ethnobotanical importance and perceptions of population status of wild-growing medicinal plants in a reserve of south-central Mexico

**DOI:** 10.1186/s13002-022-00563-3

**Published:** 2022-11-11

**Authors:** Elinor Josefina López-Patiño, Heike Vibrans, Sergio Moctezuma-Pérez, María Cristina Chávez-Mejía

**Affiliations:** 1 Instituto de Ciencias Agropecuarias y Rurales, Universidad Autónoma del Estado de México (UAEMex), El Cerrillo Piedras Blancas, 50090 Toluca, Estado de México Mexico; 2 Laboratorio de Etnobotánica, Posgrado en Botánica, Colegio de Postgraduados en Ciencias Agrícolas, Campus Montecillo, Km 36.5 Federal México-Texcoco, 56230 Montecillo, Estado de México Mexico

**Keywords:** Ethnobotany, Traditional healers, Use and cultural value, Tropical deciduous forest, Balsas River basin

## Abstract

**Background:**

The apparency hypothesis in ethnobotany (common plants are used more than less frequent ones) has been studied mostly by comparing usefulness with woody plant density, or large plants (trees) with herbs, with uneven results. Here, we explore the hypothesis for wild-growing medicinal plants, separately for different life forms. Two methodological subjects relevant for testing the hypothesis are also treated: We compare various importance indicators, including recent use, and evaluate active healers’ knowledge of plant population size. The study area was the Tenancingo-Malinalco-Zumpahuacán Protected Natural Area in central Mexico in the upper part of the Balsas River Basin, a biogeographic region with a long tradition of using wild medicinal species.

**Methods:**

Previous work on the vegetation of the protected area contributed information from 100 survey plots and a species list, which included preliminary data on the medicinal plants. Then, in 2019–2020, we held in-depth and repeated interviews with 13 traditional healers in three rural communities. They were interviewed on uses and population size of a selection of 52 medicinal species of different life forms and abundance (number of individuals in survey plots). The data were analyzed with descriptive statistics, use values and linear regression models.

**Results:**

For all species, use value correlated significantly with abundance. When separated by life forms, only herbs and shrubs/lianas showed this association, though with statistical limitations. Trees did not, perhaps because some of the most useful trees have been overcollected. We found a good correlation of recent use with frequency of mention and most other importance indicators; the correlation was weakest for number of uses. Also, active healers had a good estimation of population of their collected species.

**Conclusions:**

The apparency hypothesis should be studied separating life forms to reduce the influence of this variable. To measure importance for the study of this hypothesis, the data show that frequency of mention is a good indicator and correlated with actual use. Also, local plant users’ appreciations of population size are quite accurate in the aggregate and may be more efficient than costly vegetation surveys.

**Supplementary Information:**

The online version contains supplementary material available at 10.1186/s13002-022-00563-3.

## Background

In Mexico, between 3000 and 5000 species of plants are used for medicinal purposes [1]. Many of these species are collected from the wild, and a few are collected commercially on a large scale for markets around the country [2–4], sometimes in detriment of these wild-growing populations [2, 4]. This rich reservoir has been studied from many different angles; however, the ecological apparency/availability hypothesis [5, 6] has not been widely tested as an explanation for medicinal plant selection in Mesoamerica [7–9].

The ecological apparency hypothesis was originally proposed by Feeny (1976) and Rhoades & Cates (1976) [10, 11] and first tested in ethnobotany by Phillips & Gentry (1993) [12]. It proposed that highly visible and common (apparent/available) plants are more subject to different selective factors, particularly from herbivores, than less visible and rarer (non-apparent) species [5, 13]. Trees, shrubs or large herbs with longer life cycles were generally considered apparent, and small plants or herbs with shorter life cycles non-apparent [5]. The hypothesis predicts that the more apparent plants will have costlier defensive compounds than less apparent plants. However, the apparency hypothesis has been largely discarded, a meta-analysis failed to find evidence [7]. Rather, defensive compound production is linked to resource availability—fast-growing plants have fewer defenses than slower-growing ones [13].

Thus, the explanations have shifted: Slower-growing plants invest in more and more energy costly compounds, such as lignins and tannins. Fast-growing plants use smaller, less costly compounds for defense, such as alkaloids and terpenoids [5], which may, however, have important physiological effects [5, 13]. This explains why in many regions of the world, most medicinal plants are weedy or cultivated [7, 8]. These hypotheses have been incorporated into ethnobotanical theory as medicinal plant collectors act in some way as herbivores [8], but with the opposite aim: they seek plants with defensive compounds.

There is also a cognitive aspect. People have more opportunity to encounter, experiment with and exchange information about common plants. Phillips & Gentry (1993) [12] proposed a general positive relationship between commonness (abundance and accessibility) and the usefulness of a species. They consider that “Increasing species apparency (abundance, for example) increases their relative importance (measured by use value).”

Most studies exploring this apparency hypothesis with importance indicators were not focused on wild-growing medicinal plants, but on all uses, including timber, construction and firewood and food [6, 12, 15, 16]. As Albuquerque (2019) states, “In general, robust evidence has been obtained favoring the idea of apparency. However, the robustness depends on the utility domain considered.” Studies that include timber as a main category tend to conform with the hypothesis. If only medicinal plants were studied, and apparency calculations were based on trees being apparent and smaller plants not, no relationship was found [17]. We found no studies that distinguish apparency within life forms (trees, shrubs or herbs) or whether more common herbs are also more used as medicinals [7, 18, 19].

Recently, a much more holistic approach—considering relative overall availability, but also the maintenance of redundancy—has been proposed and documented by Albuquerque et al*.* [17]. However, the relationship between relative importance of a medicinal species and abundance in the wild-growing vegetation is still not well studied in other regions and vegetation types.

Usefulness or importance of a medicinal species has various components [12, 20], and studies do not always distinguish them clearly. Criteria may be the quantity collected, the economic value, the frequency of the illnesses treated with the plant in the population, the specificity of some plants used for treatment of certain illnesses or their versatility [21, 22]. Recently, ethnobotanists have often used versatility (the number of uses) as a criterion for estimating importance. For example, the high versatility of *Gliricidia sepium* as a medicinal and timber species supported a recommendation for priority use in restoration [23]. Very useful species with several uses are often taken into cultivation [24] and no longer collected from the wild.

This present study focuses on the relationship between various importance indicators (versatility, frequency of mention, cultural value by interviewees and a composite use value index) with an indicator of actual use, the number of people who reported use in the last year. Though there have been some comparisons, they are generally within the commonly used categories of ethnobotanical interviews (frequency of mention, number of uses, weighted importance in free lists, etc.) [24, 25]. We also compared actual use with the general use value (based on number of mentions and uses). Actual use is an important and underemployed indicator.

We also ask whether the perception of local users of wild plants, in this case traditional healers using medicinal plants, could be a guide for evaluating plant population dynamics, particularly decline [9, 20, 26–28]. The reliability of this perception is important, as one-time vegetation surveys only capture one point in time, while users have a historical perspective. Also, interviews are often less resource-intensive than comprehensive vegetation studies. Apparently, this has not been tested quantitatively for medicinal plants [2, 9]. The indicator for this knowledge in this study is the correspondence of the interviewee’s perception of present-day population size and the data on plant frequency obtained from vegetation surveys.

The Balsas River Basin in Mexico has a long tradition of providing wild-growing medicinal plants for the medicinal plant trade [4, 29], and it is also very biodiverse [30, 31]. In some rural and indigenous areas of the Balsas Basin, medicinal uses are “really significant in the population” [2, 4, 32]. The region was and is well suited for analyzing the relationship between people and medicinal plants. Our study area, the Tenancingo-Malinalco-Zumpahuacán Protected Natural Area, located in the southern part of the Federal State of Mexico, belongs to the upper part of the Balsas River Basin and is part of the cultural region of Mesoamerica [2, 29, 32]. Rural populations of Nahua origin use this reserve [33, 34]. The first author had led a previous study of the vegetation of the reserve and had access to detailed data on the plant cover, based on 100 survey plots; the flora comprising 1704 species is documented [14].

In summary, we study the relationship between the abundance of wild-growing plant species and their importance as medicinal plants, separately for three life forms, expecting to find a positive relationship between commonness of a species and its local importance. We also compare various indicators of cultural importance of medicinal species and explore the possibility of using local users’ perceptions of plant populations as a guide for their size. It is based on interviews of active traditional healers, on a selection of medicinal plants, as well as on a previous, in-depth study of the reserve’s vegetation.

## Materials and methods

### Study area

**Fig. 1:**
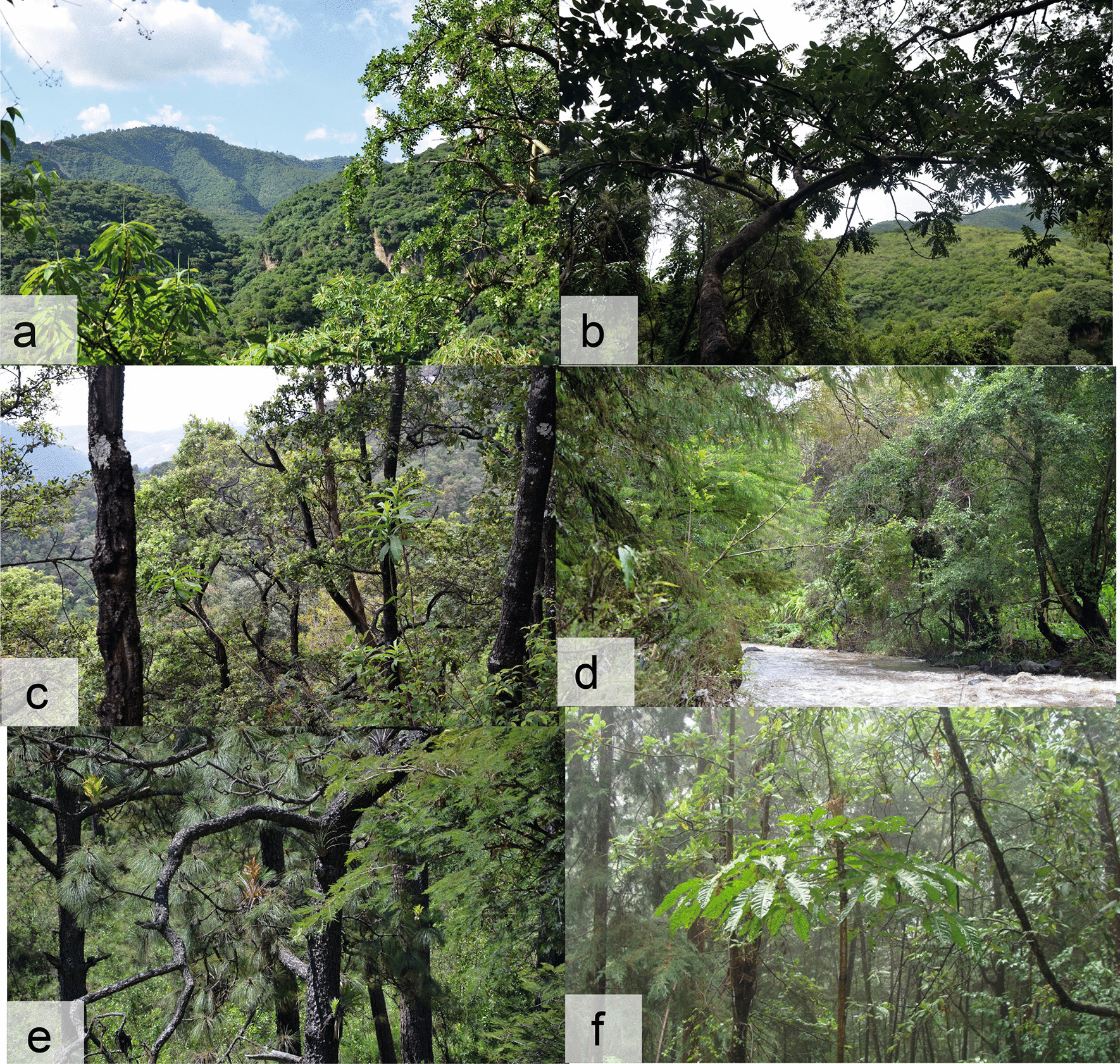
Vegetation types in the Tenancingo-Malinalco-Zumpahuacán PNA. **a** Tropical deciduous forest, **b** tropical sub-deciduous forest, **c** oak forest, **d** gallery forest, **e** pine-oak forest, **f** cloud forest

The Tenancingo-Malinalco-Zumpahuacán Protected Natural Area (TMZ-PNA) in the southern part of Mexico State (19° 5′ 0″–19° 0′ 0′′ N and − 99° 40′ 0′′ to 99° 30′ 0′′ W [13]; 1600–2700 m) is part of the Balsas River Basin and covers an area of 25,966 ha [14]. Tropical deciduous forest covers 38% of the Protected Area, tropical sub-deciduous forest 28%, temperate oak forest 9%, pine-oak forest 7%, gallery forest 13% and cloud forest 5% [14] (Fig. 1); the temperate forests are found in the northern part, from about 2000 m upward.

From 2007 to 2013, the first author co-led the project “Ecological, floristic and ethnobotanical study in the Tenancingo-Malinalco-Zumpahuacán Protected Natural Area” (registration FE012 in the National Commission for the Knowledge and Use of Biodiversity CONABIO and the Forest Protection Service of Mexico State PROBOSQUE; collection permit SEMARNAT SGPA/DGSV/05608). We collected 7187 botanical specimens, deposited in the Eizi Matuda Herbarium (CODAGEM) of the Autonomous University of the State of Mexico. The ethnobotanical part of this study documented 489 useful species, of which 234 were medicinal. We also obtained species richness and abundance data separately for three life forms (herbs; shrubs or lianas; and trees) from 100 survey plots for a total of 300 samples, located preferentially to represent different ecological conditions, particularly forested areas. The plots were 50 × 50 m for trees and, nested within these, 15 × 15 for shrubs/lianas and 2.5 × 2.5 m for herbaceous plants [36].

### Informant selection

**Fig. 2:**
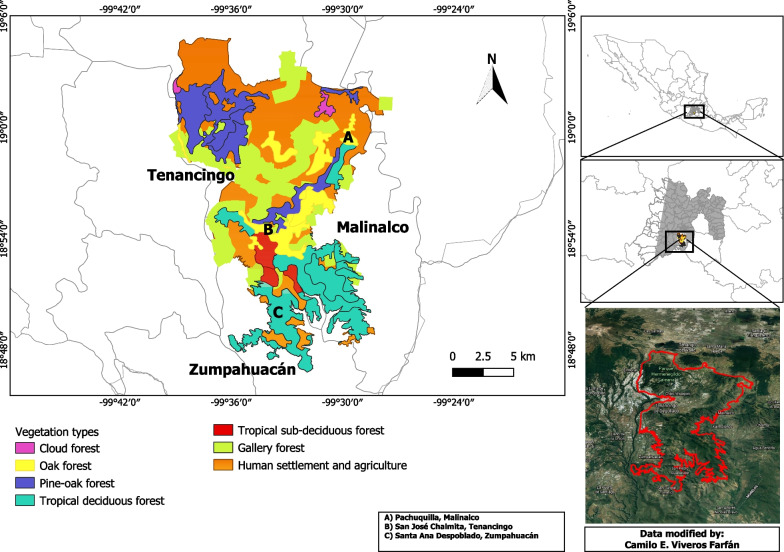
The Protected Natural Area, Tenancingo-Malinalco-Zumpahuacán (TMZ-PNA)

We chose three rural localities that were well collected during the floristic project: Pachuquilla in the municipality of Malinalco, San José Chalmita in the municipality of Tenancingo and Santa Ana Despoblado in Zumpahuacán (Fig. 2). These communities did not have health care centers nor transportation services that is less external influence, but also had poverty and poor access to public health systems.

The communities were approached in December 2019 and January 2020 through local authorities (delegates in the Mexican municipal governance system). We met with the authorities of each community, explained the purpose of the work research and requested their consent to apply the questionnaires. They also recommended the traditional healers in their community for the interviews. We asked permission and all (thirteen) were willing to collaborate; the literate persons (mainly men) signed an informed consent form issued by the Bioethics Committee of the Instituto de Ciencias Agropecuarias y Rurales of the Universidad Autónoma del Estado de México. The others gave only oral consent. The healers had different specialities: midwives [7], bonesetters [2], traditional healers [2] and collectors of medicinal plants [2], one of whom also gave massages (“sobadora”).

### Species selection

For the interviews, we selected 52 medicinal species of the TMZ-PNA (Additional file 2). The number was limited in order to avoid fatigue of the interviewees. The selection was based on the following criteria: 1. frequently mentioned as medicinals in the previous project; 2. wild-growing (some weedy), not cultivated; 3. native; 4. belonged to different botanical families and/or groups; 5. belonged to different life forms (trees, shrubs/lianas, herbs); 6. some rare in nature or first records of medicinal species for Mexico State; and 7. different distribution types in the study area (wide or restricted). For these species, we had data on distribution, vegetation type, frequency, number of individuals (from the previous survey plots), biogeographic data (particularly endemism to the Balsas Basin) and life form (herbaceous plants, shrubs and lianas and trees). “Abundance” is used to refer to the number of plant individuals counted in all survey plots.

The 52 species belonged to 48 genera and 43 families. Twenty species were herbaceous, 21 trees and 11 shrubs/lianas; 45 were native and 7 endemic to the Balsas River Basin (See Additional files 2 in Results). The most species-rich families in the list were Asteraceae, Convolvulaceae, Euphorbiaceae and Lamiaceae. The genera with more than one species were *Tagetes* [2], *Euphorbia* [2] and *Salvia* [2].

### Interviews

We interviewed 13 traditional healers (9 women and 4 men) ranging in age from 30 to 83 years. All had some distrust of Western medicine. In two of our localities, traditional medicine was still widely practiced. In San José Chalmita, Tenancingo, local authorities had prohibited traditional midwifery and medicinal plant use, but the latter was disregarded by the population. Two informants indicated that Zumpahuacán was a well-known reference for traditional medicine, and people, particularly women, would come from the outside to learn and collect plants for about a year with some of the healers.

The nine women interviewed were between 52 and 83 years old. Eight had been midwives. (Informal midwifery was now discouraged in part of the region.) One was considered an expert on medicinal plants and collected them non-commercially but traded small amounts locally. Seven sold medicinal plants locally in markets and one to nearby cities. The women interviewed were housewives and also gardened and collaborated with farmwork. Four were backstrap weavers and made garments such as shawls for sale. Other sources of income were fruits from their gardens and homemade tortillas. Two women were literate.

The four men interviewed, between 30 and 79 years of age, were all full-time practitioners of traditional medicine. They could all read and write; three, including the youngest ones, completed primary school, and all had cellphones. One was a non-practicing lawyer. Two had steam baths (*temazcal*) for the local and regional population, which was their main source of income. One of the traditional healers said he had given up farming and ranching for his healing activities; he also sold medicinal plants in the municipalities of Toluca, Metepec and Tenancingo. Three were dancers in ceremonies of pre-Hispanic origin, to celebrate the changing of the seasons.

We asked 23 questions on each plant with the aid of a photoherbarium (see Additional File 1) and also collected some basic sociodemographic data. We asked for local names, ways and frequencies of use, sources, ecological habitat and population size data, as well as the perception of the interviewees on the status of the populations (1: rare, 2: moderate, 3: abundant, 4: very abundant, 5: dominant) and their long-term tendencies, as well as causes of decline or increase.

## Data analyses

### Use value

We estimated two indicators of use value. One was the average of the number of medicinal uses (illnesses treated by the species) mentioned by every informant. This “use value” was based on the hypothesis and general observation that more important plants have more uses (Rossato et al*.*, 1999, adapted from Phillips and Gentry, 1993) [12, 31].

UV = ∑ Ui /n, where UV = total use value of species s; Ui = number of reports for each species by each informant i; and n = total number of informants.

The second indicator was based on the proportion of informants who cited the plant as medicinal (which we call “cultural significance,” but is labeled “significance level” in our source, TRAMIL) [37]. Thus, if a species was mentioned by 6 of the 13 interviewees, the cultural significance would be 46. Germosen-Robineau (2014) has worked on pharmacopoeias and, after conducting several studies on medicinal plants, found that species with a frequency of mention equal to or higher than 20% can be considered significant in terms of their cultural acceptance [37]. We adopted this cutoff point.

### Generalized linear model

The independent variable was abundance of trees, herbaceous plants and shrubs, and the dependent variable was their use value [38]. The generalized linear model was used to test the ecological apparency hypothesis [7, 39]. For data processing, the regression model 1 was applied [39–42]. The Shapiro–Wilk test was applied to determine the distribution of the data. Data did not show a normal distribution. Therefore, outlying data were eliminated so the remaining data followed a normal distribution. All data were processed in R, using the DHARMa package [44]. Outlying data were considered as those that had the minimum number of informants. Following this, the general linear model 2 was applied to the transformed variables [43]; data were logarithmized so that its distribution was symmetric, and its relationship became curvilinear. The general linear model 3 model was chosen since the family that obtained a good normality in the error distribution for our data was the Gaussian, with the minimum Akaike information criterion value (AIC).

### Statistical analyses

We calculated linear regressions with general linear models and the corresponding scatter plots for the following datasets [44, 45]:

1. Abundance vs. use value, for all species and separately for each life form.
2. The perception of population size by the informants vs. abundance data from the surveys.
3. Frequency of mentions vs. recent use (used last year).
4. Recent use vs. number of uses per species.
5. Recent use vs. use value.

## Results

### The medicinal plants and their importance

Each healer recognized 80–100% of the selected plants and used between 56 and 94% as medicinals. Every species was known as a medicinal to at least one of the interviewees. Nineteen species were mentioned by all healers; that is, they had a cultural significance of 100.

The scientific and common names of the selected species, the use values, values of cultural significance, the types of vegetation they inhabit, the frequencies of mention, the ailments for which they were indicated and the parts of the plants used are shown in Additional File 2. Fifty-two common names were recorded, and 34 of the highest use values and the frequency of mentions were found for *Tagetes lucida* (pericón), *Barkleyanthus salicifolius* (jarilla), *Amphipterygium adstringens* (cuachalalate), *Bursera copallifera* (copal), *Urtica dioica* (ortiga), *Lippia bicolor* (rosa de castilla), *Dorstenia contrajerva* (contrayerba), *Justicia spicigera* (muitle) and *Casimiroa edulis* (zapote blanco). Most species [49] had cultural significance values above 20%; the exceptions were *Dioscorea galeottiana* (cabeza de brujo) (15.3%) and *Bidens odorata* (mozote) (7.7%). *Lippia bicolor* medicinal use was a new report for Mexico.

### Ecological apparency

**Fig. 3:**
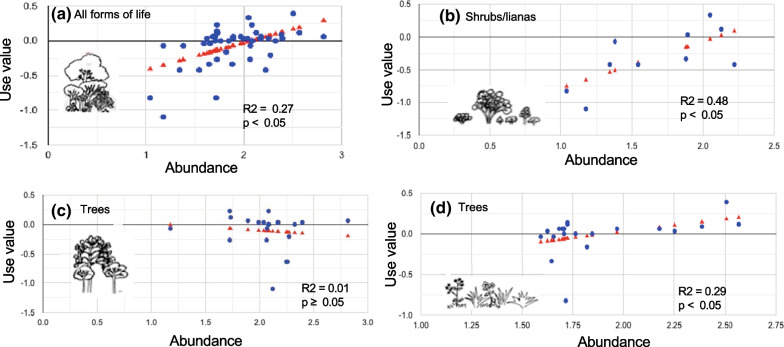
Relationship for abundance and use value for (**a**) all life forms, (**b**) herbs, (**c**) trees and (**d**) shrubs/lianas. ^·^Observations ^σ^prediction line. * y-axis: use value is the value obtained for this calculation by species. x-axis: abundance is the number of individuals per species in the vegetation surveys

**Fig. 4:**
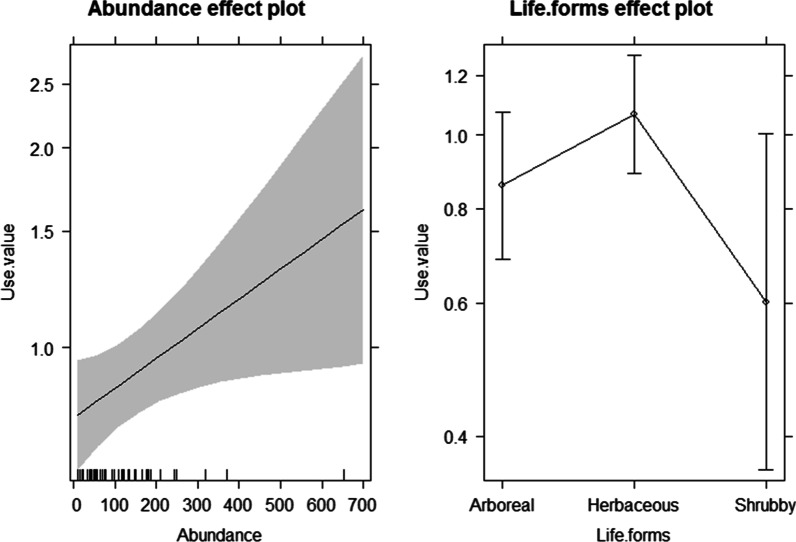
Relationship for abundance and use value for: trees, herbs and shrubs/lianas

Important medicinal species were likely to be common plants; that is, we found a positive relationship between ecological apparency (as measured by abundance) and importance indicators of medicinal use. The results of the linear regression model showed a highly significant relationship between abundance and medicinal use if all life forms were included (Table 1, Figs. 3 and 4), though with a high variance due to the heterogeneity of the data.

**Table 1:** Relationship of species abundance, life form and importance as medicinal plants, analyzed with a linear regression model (analysis of variance of the regression)

	Source	DF	Sum of Square	Mean square	Β	F Statistic	*P*-value
All forms of life	Regression	1	1.0543	1.0543	0.39, *p* < 0.001	11.8333 (1,49)	0.0012
	Residual	49	4.3656	0.0890			
	Total	50	5.4199	0.1084			
Herbs	Regression	1	0.6403	0.6403	0.54, *p* < 0.001	7.9193 (1,19)	0.0110
	Residual	19	1.5363	0.0808			
	Total	20	2.1767	0.1088			
Shrubs/liana	Regression	1	0.8144	0.8144	0.71, *p* = 0.026	7.4322 (1,8)	0.0260
	Residual	8	0.8766	0.1096			
	Total	9	1.6911	0.1879			
Trees	Regression	1	0.1079	0.1079	− 0.41, *p* = 0.170	2.0459 (1,18)	0.1697
	Residual	18	0.9494	0.05274			
	Total	19	1.0573	0.05565			

* An outlier, *Heliocarpus parvimontis,* a tree, was excluded from the regression model

If separated by life forms, we also found a positive and significant relationship for herbs and shrubs/lianas, but not for trees—for these, the relationship was slightly negative. However, for these subgroups, the data did not comply with the normality assumptions of the model, probably because of insufficient data.

### Population size perception by informants and data from survey plots

**Fig. 5:**
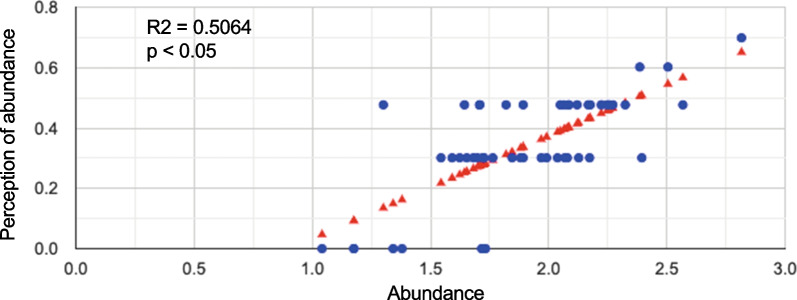
Perception of abundance by local healers and abundance data from the vegetation survey. ^·^Observations ^σ^prediction line. y-axis, perception of abundance: perception by informants obtained through interviews: (**1**) rare, (**2**) moderate, (**3**) abundant, (**4**) very abundant and (**5**) dominant. x-axis, abundance in natural vegetation: number of individuals per species in all plots of the vegetation survey

Informants estimated population size of their medicinal plants well, as shown by the linear regression model. The correlation and the P value of the relationship between their estimations and the abundance of the species in the ecological survey were highly significant (Table 2, Fig. 5), and the data complied with the assumptions of the model.

**Table 2:** Relationship between the estimations of populations size by the informants and the abundance of species in the ecological survey, analyzed with a linear regression model (analysis of variance of the regression and correlation)

Analyses between	Source	DF	Sum of Square	Mean square	β	F Statistic	*P*-value
abundance (x)/ perception of abundance (y)	Regression	1	18.7902	18.7902	0.34, *p* < 0.001	51.2951 (1,50)	3.35E-09
	Residual	50	19.2675	0.3853			
	Total	51	38.0577	0.7462

### Relative importance: relationship between various estimations

Our data allowed for an exploration of the relationships between various commonly used indicators or estimators of relative importance of ethnobotanically used species (use value based on number of uses and frequency of mention as a medicinal), and some more quantitative data (recent use). We found the following significant correlations between the independent data (that is, those that are not used for the comparison index) (Table 3):

**Table 3:** Comparison of importance indicators between a) recent use and use value, b) recent use and frequency of mention and c) frequency of mention and uses per species, all analyzed with a linear regression model (analysis of variance of the regression and correlation)

	Sources	DF	Sum of Square	Mean square	β	F Statistic	*P*-value	R^2^
Recent use/Use value	Regression	1	4.5561	4.5561	0.85 *p* < 0.001	49.4237 (1,50)	5.38E-09	0.497
	Residual	50	4.6092	0.0922				
	Total	51	9.1653	0.1797				
Recent use/Frequency of mentions	Regression	1	1.9987	1.9987	0.93 *p* < 0.001	38.5292 (1,50)	1.04E-07	0.435
	Residual	50	2.5938	0.0519				
	Total	51	4.5925	0.0900				
Frequency of mentions/Uses per specie	Regression	1	0.9069	0.9069	0.44 *p* < 0.001	15.7538 (1,50)	2.31E-04	0.2396
	Residual	50	2.8784	0.0576				
	Total	51	3.7853	0.0742				

*Recent use:* the number of interviewees who used the species in the last year. *Uses per species* is the number of illnesses for which a species is used

We found the following significant relationships:

- Recent use and use value were highly and significantly correlated; this means that species with a high use value were used currently;
- Recent use and frequency of mention were correlated significantly; that is, frequently mentioned species are likely to be used;
- Frequency of mention and number of uses were also significantly correlated, though the relationship was somewhat weaker.

## Discussion

### Current medicinal plant use

The most frequently mentioned species were well-known medicinal plants of central Mexico. Except for *Laelia autumnalis*, *Dioscorea galeottiana* and *Salvia melissodora*, all are included in the compilation of the medicinal plants of the Balsas River Basin [29]. The species with the highest use value have also been reported in other studies [4, 29, 35, 46].

An unexpected result was the existence of a kind of informal apprenticeship system in the region for medicinal plant use. This has not been reported previously for other regions in Mexico, though it probably exists.

### The ecological apparency hypothesis

The most-used species were either weedy, or woody plants of the deciduous tropical forest; native non-weedy herbs and shrubs/lianas were much less useful. The predominance of these plant types as sources of medicinal plants has been noted before [1, 2].

This study tentatively supports the ecological apparency hypothesis (more common plant species are also more commonly used) for medicinal species. It also appears to apply to subgroups based on life forms, in our case for herbs and shrubs/lianas. The lack of confirmation of the apparency hypothesis for trees may be due to the high value of the material obtained from some of them, which could have led to population decline. We do not have measured data on this, but our informants said that *Amphipterygium adstringens*, *Crescentia alata, Ternstroemia lineata* and *Eysenhardtia polystachya* (the latter also widely used as firewood) used to be more common and are collected commercially. These species are highly demanded in the national market, and there are reports of overcollection from other parts of central Mexico [4, 47]. *Persea americana* populations, which were included in the list because they occurred in apparently natural places but may have been remnants of previous human occupation, were in decline because of a disease introduced by modern varieties. *Salix humboldtiana*, a riparian species, was removed from its natural habitat to establish orchards and sugarcane plantations. However, statistical caveats apply to these results, and the subject should be explored with a larger dataset of species.

Previous work on this subject has also found mixed results, often with relatively weak, but significant correlations. Comparisons are further complicated by other works studying mainly to woody plants, and various use domains. Particularly timber trees, as mentioned in the introduction, often [26, 48], but not always [49] conform to the apparency hypothesis. Medicinal plants have been little studied [7, 8], and they used different criteria for apparency, but they did find some relationship between abundance and importance values [4].

However, their correlations are weaker than in our study; their R^2^ was 0.62, 0.59 [48] and 0.21 [50] for the life forms. They interpret this to mean that apparency is important, but not the only factor that influences plant selection. Other multidomain studies found few or negative correlations for medicinal plants [50, 51]. We suggest that our higher correlations could be due to a better dataset for this domain, and interviews with specialists, whereas the other studies interviewed the general population and had relatively few data for medicinal plants; higher correlations were also found by Christo et al. (2012) [49], who interviewed specialists on timber use. It is also possible that different vegetation types have different dynamics; perhaps the more arid vegetation types with their higher proportion of medicinals conform to the hypothesis, and more humid types do not; however, Guerra et al. [51] did not confirm the hypothesis in the Caatinga, which is similar to our dry tropical forests.

Our data can be interpreted to support both of the possible main causes of medicinal plant use, abundance and intrinsic usefulness. People use those plants that are more common and easily learned. However, there are also differences in intrinsic usefulness of ecological groups of species due to selection pressures. So, people use mainly the most common, accessible species of inherently useful groups.

We suggest that support for the apparency hypothesis, particularly for wild-growing medicinal plants, may be dynamic. This could explain the various and somewhat contradictory results obtained in different regions and vegetation types. In primary vegetation with only local uses (or in inaccessible or more distant sites), the relationship between apparency and use may be difficult to document when use is infrequent and more idiosyncratic; also, it is often a byproduct of other activities such as hunting (with some exceptions, e.g., timber use for trees). Once a vegetation type becomes accessible, medicinal plant collection is efficient, and there is an external market for some of its products, apparency should play a large role—up to a point. Once overcollection reduces the populations, the apparency hypothesis again would not be supported. So, studies should separate native and exotic species, weeds and non-weeds, commercial and local use and consider distance to the populations, collection intensity and past history [17].

Life form, just like botanical family, predicts some characteristics relevant for medicinal uses. Differing defense mechanisms leads to differences in the type of biochemical content; recovery and regeneration after damage vary substantially between herbs and trees. Also, apparency for humans depends not only on frequency, but also on size and visibility, so differences should be expected between herbs, shrubs and trees, which is shown here and should be integrated into theory. Finally, we propose that the validity of the apparency hypothesis may depend on the degree of collection pressure and thus vary over time.

### Estimations of perception population sizes from local perception

Our data show that local collectors have relatively good estimations of population size and dynamics. This means that interviews, particularly of several knowledgeable informants, can deliver useful data for management without more costly vegetation surveys, as long as interviewees are confident that their information will not be abused [27, 52, 53].

### Relationship between estimators of relative importance

Importance—the relative value that different species have for humans—is one of the basic metrics for analyzing the relationship between humans and their vegetation. A large number of methods and indices have been employed over time [12, 22, 54]. Our data allow for comparison of some widely used indicators.

Use value, frequency of mention, cultural significance and recent use were all relatively closely related. Also, people in our study cited mostly species that were in actual use, and not historical ones (which was expected, of course, as we interviewed active healers).

However, support was somewhat weaker for the common assumption that the number of uses of a medicinal plant is correlated with its importance [12, 28]. Some species, such as *Tagetes lucida*, *Tagetes micrantha* and *Barkleyanthus salicifolius*, did have high importance values and several uses*.* However, others, such as *Selaginella lepidophylla*, *Amphipterygium adstringens* or *Equisetum hyemale*, had very specific uses and were also considered important*.* Thus, number of uses as an importance indicator and part of indices should be used with some caution as it may lead to underestimation of the importance of species with only one or two uses. The relationship between number of uses and importance, particularly of wild-growing medicinals, should be explored further in other contexts.

Our data show that frequency of mention, relatively easy to obtain, is a good overall indicator of importance, and that generally, importance measured in various ways is also correlated with current use. This may be due to our species selection: We considered only native species that were obtained from natural vegetation, not the cultivated exotics that often have multiple uses [7, 55] and whose inclusion would probably have strengthened the statistical correlation between number of uses and other indicators.

### Limitations

This study has some limitations. We only included 52 species—because of constraints on the patience and attention of interviewees—which made our life form categories, and especially lianas/shrubs, rather small. The data may be susceptible to statistical error, outliers and non-normal datasets, which did occur in some data subsets. Also, the vegetation surveys were not made for this study, but rather for a vegetation description; the survey plots were placed preferentially and mostly in forested areas. Also, many other factors influence medicinal plant use—such as prevalent illnesses, accessibility and available alternatives. Plant populations can vary under different collection pressures, which may also change over time for other reasons.

However, we consider the relatively large number of vegetation plots and interviewed healers, repeated interviews and a close relationship with the interviewees, as well as the floristic knowledge of the first author compensate for some of these shortcomings. Despite the multiple factors that influence medicinal plant use, we still found some signal supporting the relationship between frequency and importance in the data. Thus, we hope to encourage other studies to look at apparency within life forms to confirm or refute the thesis that medicinal plant use is also—among other factors—influenced by the commonness of the species.

## Conclusions

Apparency, ethnobotanical importance and population trajectories are interrelated subjects that are mostly studied separately. Here, we analyzed a dataset on plant frequency and expert opinions on medicinal species from these three perspectives, to answer some theoretical and methodological questions. However, the results also illustrate the connections between these concepts.

The ecological apparency hypothesis explains part of the species selection by traditional healers in our study area. In contrast with previous studies that involved whole floras or only trees, the results show that ecological apparency and usefulness may be correlated if the data are separated and analyzed by life form. This separation should be encouraged in the future and analyzed with larger datasets. The lack of correlation in trees may have a simple explanation (e.g., overexploitation of very useful species through destructive harvesting); this was backed by observations of the interviewees. Different life forms may have different dynamics. More data are needed from other regions and vegetation types.

The number of mentions was an efficient indicator of importance, and closely related to actual (recent) use. In contrast with previous studies, the relationship of number of uses with number of mentions or recent use was relatively weak. Combining the number of uses with the number of mentions in an index improved the correlation with recent use only very slightly. The number of uses is often employed as a general indicator of importance, but the dynamics may be different in wild-growing native species than in garden plants and exotics.

Finally, we confirm that local users of biodiversity were good judges of population sizes and conservation status of the plants they use. They are also able to inform on the dynamics of the populations.

## Supplementary Information

**Additional file 1.** Species' use value and abundance.

**Additional file 2.** Plant species database.

**Additional file 3.** Local perception of population sizes.

## Data Availability

All data generated and analyzed during this study are included in Additional files 1, 2 and 3. The pictures that appear in the article were taken by one of the authors, Elinor Josefina López-Patiño. The vegetation map of the Natural Protected Area was elaborated by Camilo Viveros Farfan.

## Abbreviations

TMZ-PNA: Tenancingo-Malinalco-Zumpahuacán protected natural area
PNA: Protected natural area
